# Risk for second bladder and rectal malignancies from cervical cancer irradiation

**DOI:** 10.1002/acm2.13274

**Published:** 2021-05-21

**Authors:** Michalis Mazonakis, Efrosyni Lyraraki, Maria Tolia, John Damilakis

**Affiliations:** ^1^ Department of Medical Physics Faculty of Medicine University of Crete Crete Greece; ^2^ Department of Radiotherapy and Oncology University Hospital of Iraklion Iraklion, Crete Greece

**Keywords:** 3D‐CRT, cervical cancer, second malignancies, VMAT

## Abstract

The objective of this study was to estimate the risk of developing second malignancies to partially in‐field organs from volumetric modulated arc therapy (VMAT) of cervical cancer and to compare the above risks with those from the conventional three‐dimensional conformal radiotherapy (3D‐CRT). Seventeen consecutive patients with uterine cervix carcinoma were selected. VMAT and 3D‐CRT plans were generated with 6 and 10 MV photons, respectively. The prescribed tumor dose was 45 Gy given in 25 fractions. Differential dose‐volume histogram data from the treatment plans were obtained for the partially in‐field organs such as bladder and rectum. These data were used to estimate the patient‐specific lifetime attributable risk (LAR) for bladder and rectal cancer induction with a non‐linear model based on a mixture of plateau and bell‐shaped dose–response relationships. The estimated risks per 10000 people were compared with the baseline risks for unexposed population. The patient‐specific rectal cancer risk estimates from VMAT were significantly lower than those from 3D‐CRT (*P* = 0.0144). The LARs for developing bladder malignancies from VMAT were significantly high compared to those from conventional irradiation (*P* = 0.0003). The mean difference between the patient‐specific LARs for radiation‐induced bladder and rectal malignancies as derived from 3D‐CRT and VMAT plans was 6.6% and 2.0%, respectively. The average LAR for developing bladder and rectal malignant diseases due to VMAT was 9.2 × 10^‐4^ and 43.7 × 10^‐4^, respectively. The corresponding risks following 3D‐CRT were 8.6 × 10^‐4^ and 44.6 × 10^‐4^. These average risks showed that pelvic irradiation increases the baseline probability for cancer induction by 12.6‐19.1%. The differences in the second cancer risks associated with the VMAT and 3D‐CRT for cervical cancer were found to be small. Both treatment techniques resulted in considerable increased probabilities for developing bladder and rectal malignancies relative to those of unirradiated population.

## 1 INTRODUCTION

Cervical cancer is the fourth most frequent malignant disease and, also, the fourth main cause of cancer mortality in females globally.^1^ The 5‐year survival rate of this type of cancer is 66.1% and it reaches to 91.8% for localized disease stage.^2^ Radiation therapy plays a major role in the management of the uterine cervix carcinoma.^3^ Previous studies reported that the therapeutic irradiation of this gynecological cancer is associated with an increased probability for developing second malignancies to surrounding heavily exposed sites such as urinary bladder and rectum.^4^, ^5^, ^6^ The above results were derived from patients irradiated before 2001 without advanced treatment delivery and planning approaches.

The use of intensity modulated techniques is currently recommended for external‐beam radiotherapy of cervical cancer.^7^ The intensity modulated radiation therapy (IMRT) and volumetric modulated arc therapy (VMAT) may lead to a significant reduction of the acute toxicity compared to that from conventional pelvic irradiation.^8^ However, the application of IMRT and/or VMAT in clinical practice always creates a concern about the second cancer risk magnitude due to the long delivery times and the large volume of exposed normal tissues in respect to those related to three‐dimensional conformal radiation therapy (3D‐CRT).^9^ To the best of our knowledge, there is only one report dealing with carcinogenesis from IMRT for cervical cancer.^10^ They found that IMRT results in a higher cumulative cancer risk than 3D‐CRT.

The VMAT for cervical cancer has gained popularity the last decade due to the decrease of the treatment delivery time and monitor unit (MU) usage in comparison with IMRT.^11^, ^12^, ^13^ No attempts have been made to assess the probability for second cancer development in patients subjected to VMAT for gynecological carcinomas. This study was conducted to estimate the risks for developing second malignancies to critical partially in‐field organs such as bladder and rectum following VMAT for cervical cancer and to compare the aforementioned risks with those resulting from conventional 3D‐CRT.

## 2 MATERIALS AND METHODS

### 2.1 CT scanning and contouring

**Fig. 1:**
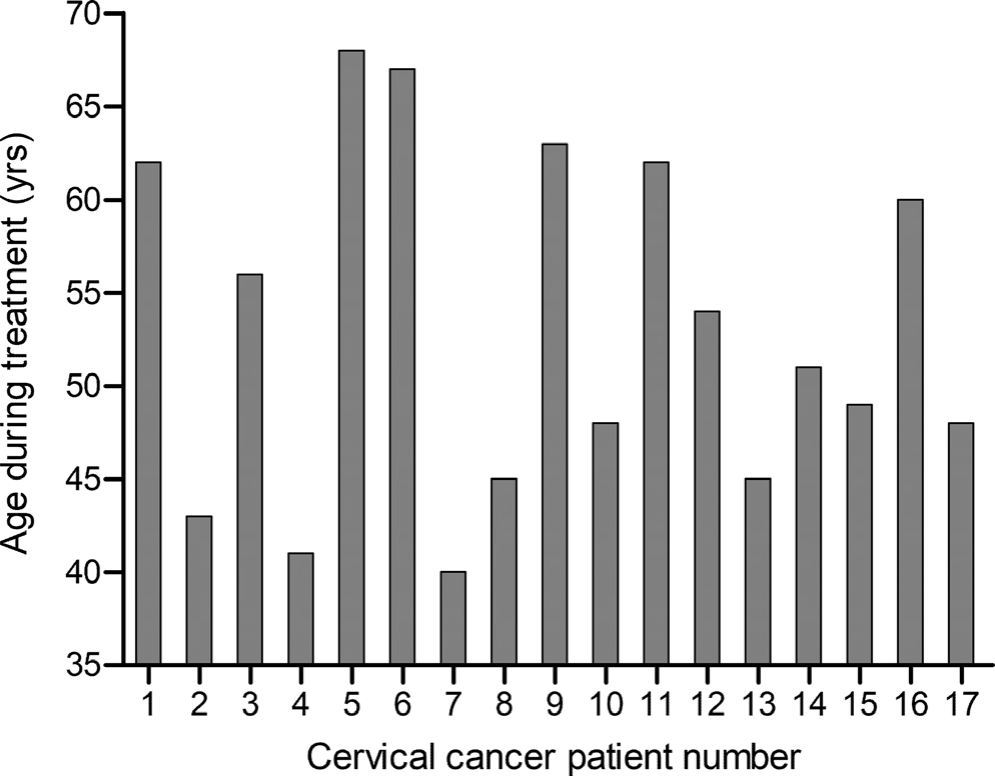
Age of the cervical cancer patients during external‐beam radiation therapy.

Seventeen consecutive patients with localized carcinoma of the uterine cervix were included in this work. The patient’s age ranged from 40 to 68 yrs old with a mean age of 53.1 ± 9.2 yrs (Fig. 1). The study participants underwent a planning computed tomography (CT) examination in supine treatment position. The CT slice thickness was 5 mm without a gap. All CT datasets were transferred to Monaco system (Elekta AB, Stockholm, Sweden) for contouring and planning. The RTOG‐0418 guidelines^14^ were used to define the targets and organs at risk (OARs) on the CT scans acquired with a full urinary bladder and an empty rectum. The clinical target volume (CTV) comprised the upper 3.0 cm of vagina and paravaginal soft tissue lateral to the vagina. The internal, external and common iliac lymph nodes, and presacral nodal regions were also included within the CTV. A 7‐mm margin was applied to uniformly expand the CTV to the planning target volume (PTV). The bowel, rectum, urinary bladder, and femoral heads were manually delineated on the CT scans as the organs‐at‐risk (OARs). The study protocol was approved by the ethics committee of our institution. A written informed consent was obtained by the participants.

### 2.2 3D‐CRT and VMAT planning

For each patient, treatment plans were generated with 3D‐CRT and VMAT for delivery on a medical linear accelerator (Infinity, Elekta AB, Stockholm, Sweden) emitting 6 and 10 MV photons. The PTV prescribed dose was 45 Gy given in 25 daily fractions of 1.8 Gy. The 3D‐CRT plans involved 10 MV photon beams. The beam arrangement consisted of an anteroposterior, a posteroantrior and two lateral opposed treatment fields at the following gantry angles: of 0°, 90°, 180°, and 270°. The beam weight was optimized for each separate plan in order to achieve a homogeneous dose distribution within the PTV and reduce the radiation dose to surrounding structures. The planning aim was to cover at least 95% of the PTV with the 95% of the prescribed dose.

The VMAT plans were created with 6 MV photons. Two coplanar full arcs rotating in clockwise and anticlockwise directions were used. The segment width was set to 1.0 cm^15^ and the increment of gantry was 30°.^16^ All VMAT plans were designed to satisfy the dose constraints introduced by the RTOG 0418 for modulated radiotherapy.^17^ These constraints are summarized in Table 1. Furthermore, the maximum PTV dose was kept below 49.5 Gy whereas less than 1% of the target volume received a dose smaller than 93% of the prescribed dose. No more than 1% or 1 cm^3^ (whichever is lower) of the healthy tissues outside PTV absorbed a radiation dose exceeding 110% of the tumor dose.

**Table 1:** Dosimetric constraints of the planning target volume (PTV) and surrounding critical organs applied for VMAT planning.

Structure	V_i_ ^a^
PTV	V_45_ ≥97%
Bowel	V_40_ <30%
Rectum	V_30_ <60%
Bladder	V_45_ <35%
Femoral heads	V_30_ ≤15%

^a^ The V_i_ corresponds to the structure volume absorbing a radiation dose of i Gy.

### 2.3 Estimation of the patient‐specific cancer risk

Pelvic radiation therapy for gynecological carcinomas may result in second bladder and rectal malignant diseases.^4^, ^5^, ^6^, ^10^ The above structures were characterized as partially in‐field organs in accordance with a previous study of Howell et al.^18^ The above OARs were adjacent to the target volume and they received an inhomogeneous dose distribution in the 3D‐CRT and VMAT plans of all study participants. Parts of both rectum and urinary bladder absorbed radiation doses up to that of the PTV.

Hall^19^ reported that the risk of radiation carcinogenesis is linearly related to the absorbed dose for a dose range of 0.1–2.5 Gy. Dasu and Toma‐Dasu^20^ also stated that the linear relationship exists up to doses of 1–2 Gy. The extrapolation of the linear‐no‐threshold approach to doses exceeding the above levels is not recommended.^21^ There are several non‐linear models in the literature which ignore either the fractionation or the cell proliferation effects occurring during radiotherapy.^20^ Schneider et al.^22^ introduced a mechanistic model providing site‐specific dose response relations using data obtained by A‐bomb survivors and irradiated Hodgkin’s disease patients. This mechanistic model is based on a mixture of plateau and bell‐shaped dose response relationships. The model‐based cancer risk estimates account for the tumor dose fractionation and the interfraction repair of the exposed tissues.

The above non‐linear mechanistic model was applied to estimate the cancer risk to bladder and rectum from radiation therapy for cervical cancer. The probability of carcinogenesis to critical organs has been previously assessed with this model for patients irradiated for carcinomas^23^, ^24^, ^25^ and benign disorders.^26^ The application of the mechanistic model required the knowledge of the organ equivalent dose (OED) of each OAR. Differential dose‐volume histograms were used to calculate the OED of the bladder or rectum from the 3D‐CRT and VMAT plans of each patient as follows:

(1) $$\text{OED}=\frac{1}{V_{t}}\sum_{i}V_{D_{i}}\frac{e^{‐a_{i}^{\prime }D_{i}}}{a_{i}^{\prime }R}\left[1‐2R+R^{2}e^{a_{i}^{\prime }D_{i}}‐{\left(1‐R\right)}^{2}e^{‐\frac{a_{i}^{\prime }R}{1‐R}D_{i}}\right]$$

where $V_{t}$is the total organ volume, $V_{D_{i}}$ is the organ volume receiving a dose equal to $D_{i}$, R is a factor associated with the organ‐dependent repopulation and $a_{i}^{\prime }$ is the cell killing factor. The $a_{i}^{\prime }$ was calculated using the formula:

(2) $$a_{i}^{\prime }=a+\beta D_{i}\frac{D_{f}}{D_{t}}$$

where a and β are the linear quatradic parameters, $D_{f}$ is the PTV dose of 1.8 Gy delivered in each fraction and $D_{t}$ is the total target dose of 45 Gy.

The patient‐dependent excess absolute risk (EAR) for the appearance of radiation‐induced bladder or rectal malignancies due to 3D‐CRT or VMAT for cervical cancer was estimated as follows:

(3) $$\text{EAR}=\text{OED}\beta_{\mathrm{EAR}}\;\exp\left[\gamma_{e}\left(age_{e}‐30\right)+\gamma_{a}\text{ln}\left(\frac{age_{a}}{70}\right)\right]$$

where $\beta_{\mathrm{EAR}}$ is the initial slope at low doses of the dose‐response curve for a Western population, $\gamma_{e}$ and $\gamma_{a}$ are the age modifying parameters for each organ of interest, $age_{e}$ is the age of the female patient during radiation therapy and $age_{a}$ is the attained age. The lifetime attributable risk (LAR) for second cancer induction was estimated with the formula:

(4) $$\text{LAR}=\int_{{\text{age}}_{e}+L}^{{\text{age}}_{a,max}}\text{EAR}\left({\text{age}}_{e},{\text{age}}_{a}\right)\frac{S\left({\text{age}}_{a}\right)}{S\left({\text{age}}_{e}\right)}d\left({\text{age}}_{a}\right)$$

where the ${\text{age}}_{a,max}$ was taken equal to 80 years, L is a cancer‐risk free period of 5 years for the development of radiation‐induced solid tumors and $S\left({\text{age}}_{a}\right)/S\left({\text{age}}_{e}\right)$ is the probability of a healthy female to survive from $age_{e}$ to $age_{a}$ obtained by the United States life tables.^27^ The parameters, R, a, $\gamma_{e}$, $\gamma_{a}$ and $\beta_{\mathrm{EAR}}$ for the bladder were taken from the literature and they were 0.06, 0.219 Gy^‐1^, −0.024, 2.38, and 3.8/(10^4^ PY Gy), respectively.^22^ The corresponding parameters for the rectum were 0.56, 0.033 Gy^‐1^, −0.056, 6.9 and 0.73/(10^4^ PY Gy).^22^, ^23^ The LAR estimates were expressed as the risk per 10000 people.

### 2.4 Estimation of the average cancer risk

The average OED_av_ of bladder and rectum was found from the 3D‐CRT and VMAT plans of all patients. The calculated OED_av_ was employed to estimate the respective average lifetime risk (LAR_av_) for the appearance of radiotherapy‐induced bladder and rectal malignancies using the equations of the previous subsection. The LAR_av_ was estimated for a typical 50‐year‐old patient at the time of irradiation and an attained age of 80 yrs. The LAR_av_ was combined with the baseline risk (BR) to estimate the average relative risk (RR_av_) of carcinogenesis as follows:

(5) $$RR_{\mathrm{av}}=\left(LAR_{\mathrm{av}}+BR\right)/BR$$

Based on SEER data,^2^ the BR of a 50‐year‐old healthy female to be diagnosed with bladder and rectal malignancies in 30 yrs is 0.68% and 2.33%, respectively.

### 2.5 Statistics

The patient‐specific second cancer risk estimates related to 3D‐CRT for cervical carcinoma were compared with the assessments derived from the VMAT plans by using a Wilcoxon signed‐rank test. Differences at the level of *P*‐value of 0.05 or less were considered as significant. The Bland‐Altman statistical test was also applied to determine the percentage mean difference (MD) between the organ‐specific cancer risks estimated by the two treatment delivery techniques. The 95% confidence intervals between the risks derived from the two techniques were set to MD±1.96 SD, where SD is the standard deviation of the differences.^28^ Statistical analysis was performed using the GraphPad Prism software (Graph Pad Software Inc., CA, USA).

## 3 RESULTS

### 3.1 Patient‐specific cancer risk estimates

**Fig. 2:**
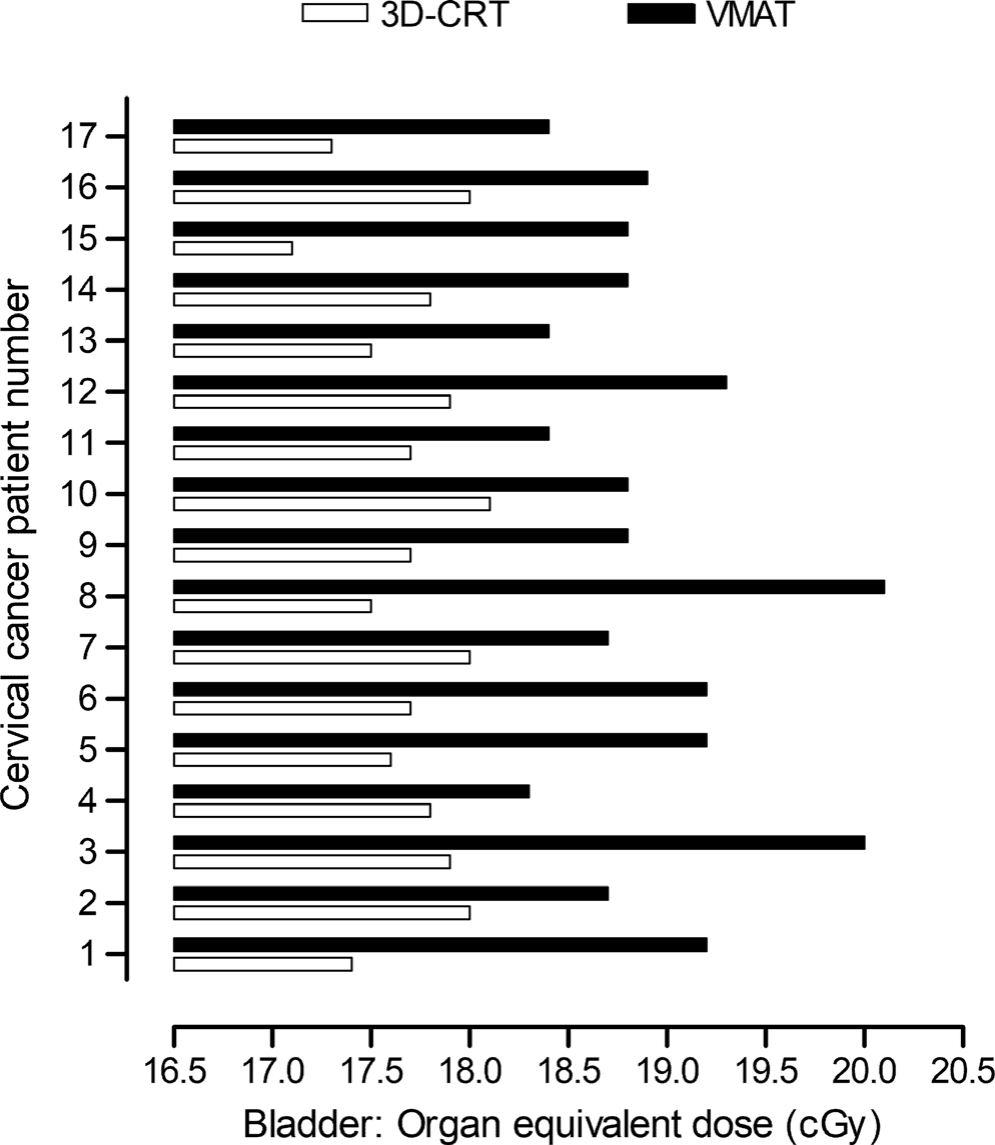
Organ equivalent dose calculations for bladder derived from the 3D‐CRT and VMAT plans of the cervical cancer patients.

**Fig. 3:**
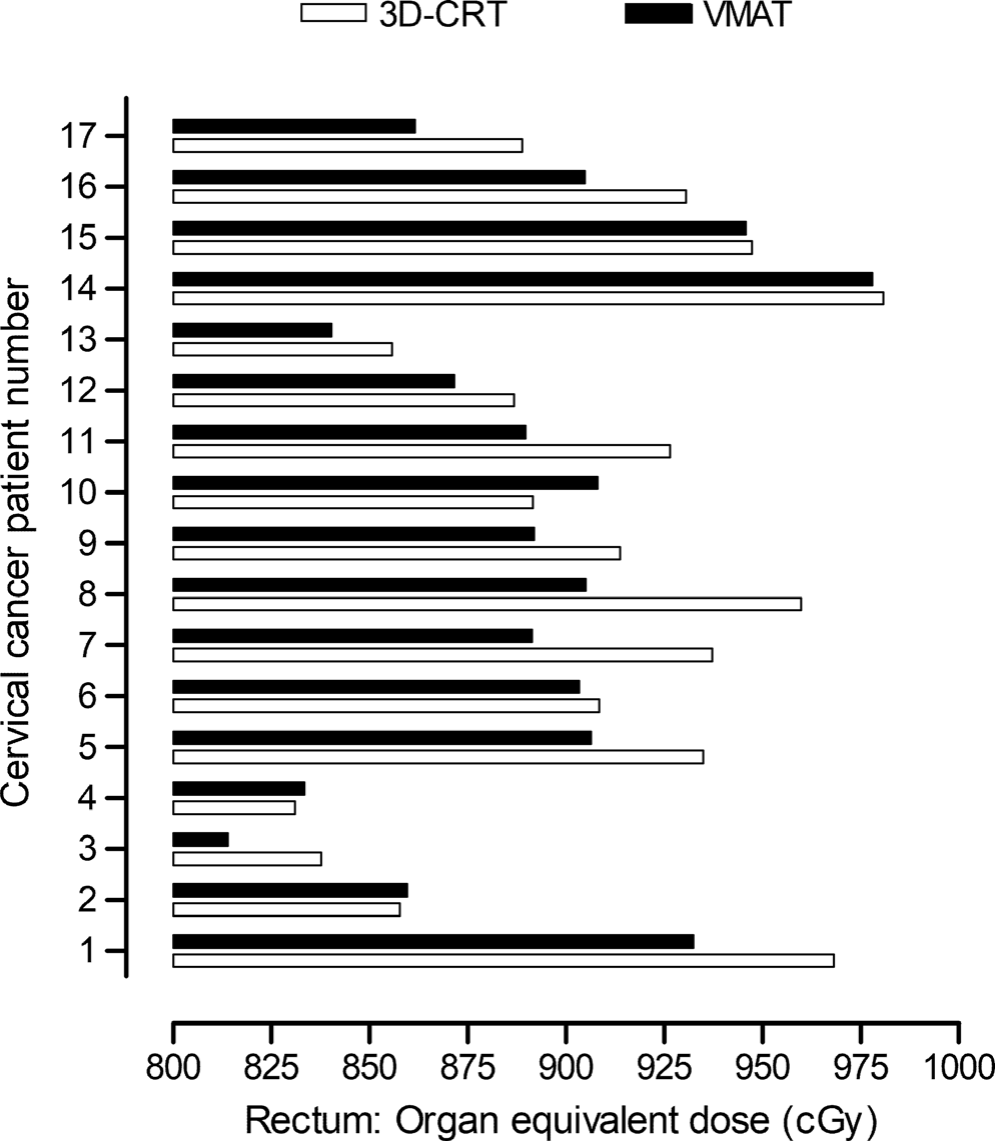
Organ equivalent dose calculations for rectum derived from the 3D‐CRT and VMAT plans of the cervical cancer patients.

The OED calculations are presented in Figs. 2 and 3. The OED range of the bladder from 3D‐CRT and VMAT was 17.1–18.1 cGy and 18.3–20.1 cGy, respectively (Fig. 2). The corresponding range for rectum was 831.0–980.7 cGy and 813.9–977.9 cGy (Fig. 3). The lifetime risks for the development of radiotherapy‐induced malignancies are presented in Table 2. The LAR range for bladder cancer induction associated with 3D‐CRT and VMAT for cervical cancer was found to be 2.3 × 10^‐4^ to 13.2 × 10^‐4^ and 2.5 × 10^‐4^ to 13.7 × 10^‐4^, respectively. The corresponding LARs for rectal malignancies were 10.3 × 10^‐4^ to 82.8 × 10^‐4^ and 9.9 × 10^‐4^ to 78.3 × 10^‐4^.

**Table 2:** Lifetime attributable risk (LAR) estimates per 10000 people for the development of second bladder and rectal malignancies derived from 3D‐CRT and VMAT plans of patients with cervical cancer.

Patient	LAR for bladder cancer (×10^‐4^)		LAR for rectal cancer (×10^‐4^)	
	3D‐CRT	VMAT	3D‐CRT	VMAT
1	4.1	4.2	20.3	19.6
2	11.8	12.2	63.7	63.9
3	6.3	7.1	27.9	27.1
4	12.6	13.0	69.3	69.9
5	2.3	2.5	10.3	9.9
6	2.6	2.8	11.4	11.3
7	13.2	13.7	82.8	78.3
8	10.6	12.1	63.5	59.9
9	3.9	4.1	17.5	17.1
10	9.4	10.0	49.4	50.3
11	4.2	4.3	19.4	18.7
12	7.1	7.7	33.8	33.2
13	10.5	11.1	56.6	55.6
14	8.3	8.7	45.2	45.1
15	8.7	9.8	49.4	49.3
16	4.9	5.2	23.0	22.4
17	9.0	9.8	49.2	47.7

**Fig. 4:**
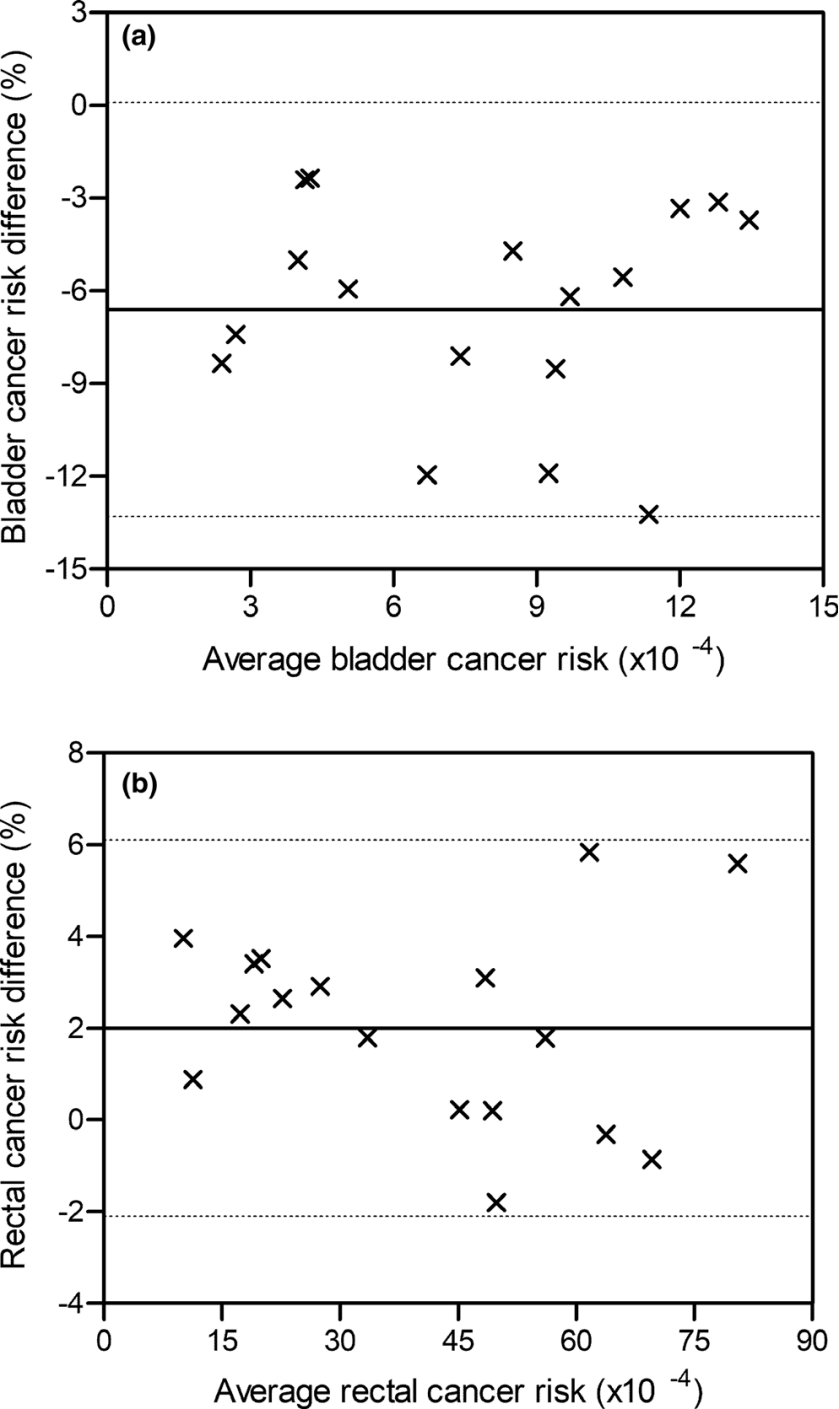
Scatter plots presenting the differences between the patient‐specific risks for developing bladder cancer (a), and rectal cancer (b) as estimated by the 3D‐CRT and VMAT plans of patients with primary carcinoma of the uterine cervix against the average risk value. The dotted lines illustrate the 95% confidence intervals and the solid line is the mean% risk difference.

The LARs for second cancer induction from 3D‐CRT were significantly different from those related to VMAT for cervical cancer (Bladder: *P* = 0.0003; Rectum: *P* = 0.0144). The use of 3D‐CRT resulted in lower bladder cancer risks than VMAT for all patients examined (Table 2). Conventional treatment led to an increased rectal cancer risk compared to that from VMAT in 14 of 17 study participants (Table 2). The Bland‐Altman scatter plots are shown in Fig. 4. The MD between the bladder cancer risk estimates associated with 3D‐CRT and VMAT was −6.6 ± 3.4% with 95% confidence intervals of 0.1% to −13.3%. The corresponding MD in the assessment of the probability for rectal cancer development was 2.0 ± 2.1%. The 95% limits of agreement were equal to −2.1% and 6.1%.

### 3.2 Average cancer risk estimates

The OED_av_ of bladder attributable to 3D‐CRT and VMAT for cervical carcinoma was 17.7 and 18.9 cGy, respectively. The corresponding quantity for the rectum was 909.2 and 890.4 cGy. The average risks are presented in Table 3. The LAR_av_ for the induction of second malignancies ranged from 8.6 × 10^‐4^ to 44.6 × 10^‐4^ by the organ of interest and the treatment delivery technique. The RR_av_ related to bladder and rectal cancer development was up to 1.135 and 1.191, respectively.

**Table 3:** Average lifetime attributable risk (LAR_av_) and average relative risk (RR_av_) for the development of second bladder and rectal malignancies following 3D‐CRT and VMAT of a 50‐year‐old cervical cancer patient.

Organ‐at‐risk	Technique	LAR_av_ (×10^‐4^)	RR_av_
Bladder	3D‐CRT	8.6	1.126
	VMAT	9.2	1.135
Rectum	3D‐CRT	44.6	1.191
	VMAT	43.7	1.187

## 4 DISCUSSION

The unavoidable exposure of healthy tissues to ionizing radiation during external‐beam radiotherapy of primary neoplasms may elevate the risk for subsequent carcinogenesis. The appearance of second tumors at distant sites from the primarily irradiated area is relatively rare. Dorr and Hermann^29^ found that almost 50% of the second tumors appear in the margin area of the treatment volume and less than 10% within the radiation fields. Welte et al.^30^ showed that 69% of the total number of second malignancies are presented at the field margins or inside the primarily irradiated region. The current study was focused on the assessment of the second cancer risk to the partially in‐field bladder and rectum due to radiation therapy for uterine cervix carcinoma. These organs, which are characterized by the high susceptibility for radiation carcinogenesis, were partly exposed to primary radiation due to their adjacent location to the treatment volume.

The probabilities for the appearance of second malignancies were estimated for 3D‐CRT with 10 MV photons and 6 MV VMAT of cervical carcinoma. The above photon energies are usually employed in our department for non‐modulated and modulated treatment of primary pelvic tumors. The neutron contribution to the exposure of critical sites due to irradiation with 10 MV X‐rays was considered as minimal.^31^ The second bladder and rectal cancer risks varied considerably by the patient’s age during treatment. Based on the risks shown in Table 2, the LAR for developing second bladder malignancies due to irradiation of a 40‐year‐old female (patient no. 7) was 5.5 times higher than that for patient no. 5 who was 68‐year‐old at the time of treatment. The corresponding ratio related to the second rectal cancer was about 8.0. The use of 3D‐CRT resulted in significantly lower patient‐specific LARs for bladder cancer induction than those from VMAT. The opposite result was found for the rectal cancer risk where VMAT significantly reduced this risk compared to 3D‐CRT. Despite the above statistical significance, the differences between the second cancer risks estimated by the two treatment techniques were small for all patients. Bland‐Altman analysis showed that the MD for the bladder and rectal cancer risk derived from pelvic VMAT and 3D‐CRT is only 6.6% and 2.0%, respectively.

The RR_av_ for developing a second malignancy to bladder or rectum after radiation therapy for cervical cancer was assessed for a typical 50‐year‐old female. The RR_av_ for rectal cancer induction due to VMAT was 1.187. This result revealed that the application of the above treatment technique may elevate the lifetime rectal cancer risk by 18.7% in comparison to the probability of unirradiated 50‐year‐old females. The corresponding increase associated with the conventional 3D‐CRT was 19.1%. The use of VMAT and 3D‐CRT was also found to give an increased probability for developing bladder malignancies in respect to unexposed people by 13.5% and 12.6%, respectively. The above baseline cancer risk increases are considerable and they cannot be ignored by physicians who follow‐up the irradiated patients.

Limited data have been published about the theoretical risk of carcinogenesis to adjacent heavily exposed organs attributable to radiation therapy for cervical cancer.^10^, ^32^ The cancer risk to the entire colon was estimated in these reports. Based on our results, the lifetime bladder cancer risks in females treated during the 5th decade of life with 3D‐CRT were 8.7 × 10^‐4^ to 13.2 × 10^‐4^. These probabilities are similar to the lifetime risks of (12–14) × 10^‐4^ for 40‐year‐old patients undergoing four‐field box irradiation of gynecological carcinomas with 6 MV photons.^32^ Zwaheln et al.^10^ showed that the 6MV‐IMRT leads to a bladder cancer risk increase of 11.4% compared to 3D‐CRT when a linear‐exponential model is used, whereas, the data analysis with a plateau model gave a decrease of 0.4%. Their results are comparable with the low differences presented in our study based on the application of a non‐linear mechanistic model. We found that VMAT increases the probability for developing bladder malignancies in respect to 3D‐CRT by 2.3%–13.2% in 95% of the irradiated patients.

The presented OEDs of bladder and rectum and the relevant cancer risks might contain uncertainties arising from the analysis of the DVHs derived from a commercial treatment planning software. Parts of the above organs‐at‐risk were located close but outside the treatment volume. Inaccuracies may exist in the out‐of‐field dose calculations generated by treatment planning systems.^33^ The results of this work may be also limited by errors in the definition of the organ‐specific parameters of the mechanistic model applied for cancer risk estimation. The quantity a/β was taken equal to 3 Gy for all tissues under investigation in accordance with the recommendation of Schneider et al.^22^ Reported experience has shown an insignificant change of the breast cancer risk with a/β values from 1 to 5 Gy.^34^ It has to be mentioned that the estimated probabilities for radiation‐induced bladder and rectal malignancies presented here solely referred to therapeutic doses. The use of image guidance procedures in clinical practice may result in small absorbed doses to these critical organs compared to those from radiation therapy.^35^ Further research is required to evaluate whether these imaging doses have an impact on the second cancer risk magnitude.

## 5 CONCLUSIONS

The use of VMAT for cervical cancer significantly reduced the probability for developing second rectal malignancies than 3D‐CRT. The bladder cancer risk from VMAT was significantly high compared to that from the conventional irradiation. However, the absolute differences in the patient‐specific probabilities for the appearance of second malignancies due to VMAT and 3D‐CRT were found to be small. Both delivery techniques led to noticeable elevated second cancer risks compared to those for unirradiated population.

## CONFLICT OF INTEREST

The authors declare that there is no conflict of interest regarding the publication of this article.

## Data Availability

The data that support the findings of this study are available from the corresponding author upon reasonable request.
